# Comparison of kinematic variables obtained by inertial sensors among stroke survivors and healthy older adults in the Functional Reach Test: cross-sectional study

**DOI:** 10.1186/s12938-015-0047-z

**Published:** 2015-05-30

**Authors:** José Antonio Merchán-Baeza, Manuel González-Sánchez, Antonio Ignacio Cuesta-Vargas

**Affiliations:** Departamento de Psiquiatría y Fisioterapia, Instituto de Investigación Biomédica de Málaga (IBIMA), Universidad de Málaga, 29071 Málaga, Spain; School of Clinical Sciences of the Faculty of Health, Queensland University of Technology, Level 6, O Block, D Wing, Kelvin Grove, Brisbane, Australia

**Keywords:** Inertial sensor, Functional Reach Test, Stroke survivors, Healthy older adults, Kinematic variables, Reliability

## Abstract

**Background:**

Balance dysfunction is one of the most common problems in people who suffer stroke. To parameterize functional tests standardized by inertial sensors have been promoted in applied medicine. The aim of this study was to compare the kinematic variables of the Functional Reach Test (FRT) obtained by two inertial sensors placed on the trunk and lumbar region between stroke survivors (SS) and healthy older adults (HOA) and to analyze the reliability of the kinematic measurements obtained.

**Methods:**

Cross-sectional study. Five SS and five HOA over 65. A descriptive analysis of the average range as well as all kinematic variables recorded was developed. The intrasubject and intersubject reliability of the measured variables was directly calculated.

**Results:**

In the same intervals, the angular displacement was greater in the HOA group; however, they were completed at similar times for both groups, and HOA conducted the test at a higher speed and greater acceleration in each of the intervals. The SS values were higher than HOA values in the maximum and minimum acceleration in the trunk and in the lumbar region.

**Conclusions:**

The SS show less functional reach, a narrower, slower and less accelerated movement during the FRT execution, but with higher peaks of acceleration and speed when they are compared with HOA.

## Background

Balance dysfunction is one of the most common problems in people who suffer stroke and it has a great impact on functional independence and on the recovery of the individual [1, 2]. The most significant physical impact on stroke patients is long-term disability, which is mainly caused by hemiparesis [2–4].

Due to postural control problems, the reduction of functional skills as well as the loss of static and dynamic stability in people with stroke, such as loss of early activation during voluntary movements, a greater sway in static standing, especially on the affected side, and decreased stability during weight change while standing [1, 2, 5, 6], which could result in an increased risk of falls [7–9].

A widely accepted clinical tool for the assessment of imbalance is the Functional Reach Test (FRT), which has been used to measure biomechanics, postural control and balance in patients who suffer from Parkinson’s disease, physical frailty, vestibular dysfunction and stroke [4, 10]. This test evaluates these variables by measuring the maximum functional reach a person can achieve in the frontal plane without losing balance, stepping, or falling. The test is a tool designed for simple, reliable, economical and portable measurement [4, 11–14].

In different fields of applied medicine, parameterization has been promoted in the execution of functional tests standardized by inertial sensors. These tests measure the health status of patients and help to establish and implement effective treatment strategies [15, 16]. Inertial sensors are instruments capable of collecting kinematic variables of any gesture or movement due to their size, portability, and reliability [17, 18]. These instruments have been used both in clinical practice as tools for feedback to improve rolling-on tests of balance and ambulation [18, 19] and in basic research, to analyze the different kinematic variables into which the gait can be decomposed [17, 20–23].

No studies were found in which the kinematic variables registered with inertial sensors located in the lumbar region and trunk during the execution of the FRT in stroke survivors (SS) and healthy older adults (HOA) are compared.

The aim of this study is to compare the kinematic registration of a balance test (FRT) with an inertial sensor placed on the trunk (L5–S1) and another in the lumbar region (T7) between stroke survivors and HOA.

A secondary aim of this study is to analyze the reliability of the kinematic measurements obtained with inertial sensors in two different body regions during the FRT.

The hypothesis of this study is that significant differences exist in the kinematic parameters recorded between SS and HOA. In addition, it is expected that the inertial sensors will be shown to be reliable tools for the kinematic recording of the FRT.

## Methods

### Design and participants

This is a cross-sectional study for which participants (n = 10) met the following general inclusion criteria: performing the Time Up and Go test in 15 s or less and being able to remain standing without assistance for more than 30 s. Specific inclusion criteria for participants with stroke were said disease as defined by the World Health Organization [24] and moderate severity (score between 0 and 49 on Barthel’s Index) [25]. Exclusion criteria were being younger than 60, limitations in walking, severe problems of communication or understanding, serious cardiovascular, respiratory, metabolic or orthopedic problems, suffering from a secondary neurological disease and failing to provide informed consent.

This study was conducted according to the principles of the Declaration of Helsinki for the protection of the rights, safety and welfare of the volunteers who participated in it. Ethical approval for the study was granted by the ethics committee of the Faculty of Health Sciences, University of Malaga.

Participants were given an information sheet, which explained in detail the development of the study, and an informed consent, which made it clear that their participation was completely voluntary and that their personal data will be protected according to the Organic Law of Protection of Personal Data 19/55.

### Functional Reach Test (FRT)

**Figure 1:**
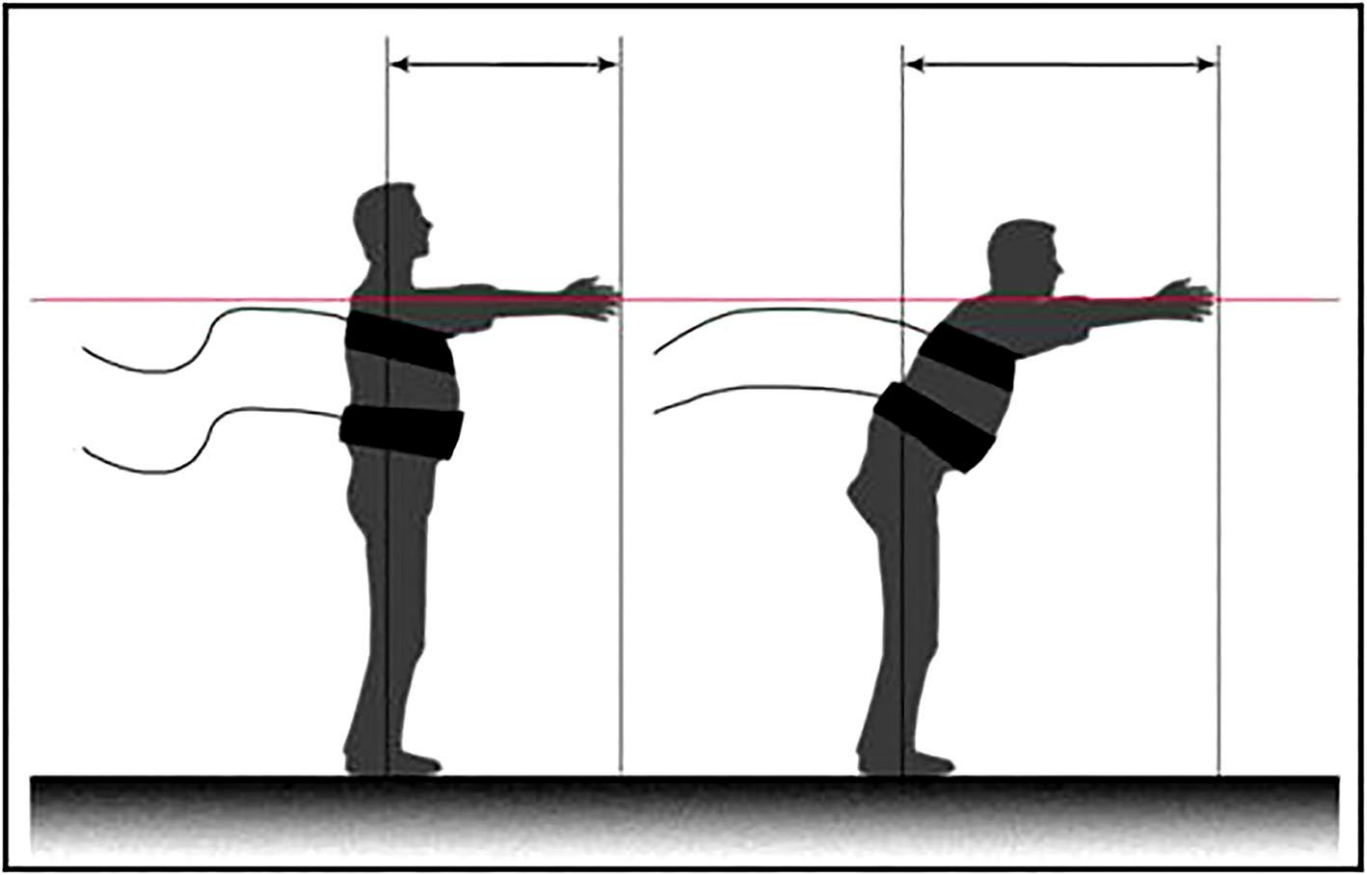
Performing of the FRT.

To implement the FRT a tape is placed on the wall, parallel to the floor, up to the acromion of the dominant arm of the subject. Then the participant is asked to position himself parallel to the wall where the tape is attached so that the axis passing through his shoulder is as perpendicular as possible to the surface thereof. Next, the participant is told that their feet should be separated at shoulder width and that he must not touch the wall during the test. In addition, participants are asked to flex the shoulder to 90° and straighten their elbows and hands; at this time, the researcher makes a mark on the tape using the metacarpal head of the third finger as a reference point. The participant attempts to reach far as possible without taking a step, lifting a heel or touching the wall. In that moment is when the second mark on the wall is made, and thereafter, the subject returns to the starting position. The distance in centimeters between the two marks is the functional range achieved by the participant [26] (Figure 1). Previous studies have shown the reliability of the FRT is 0.81 [26].

**Figure 2:**
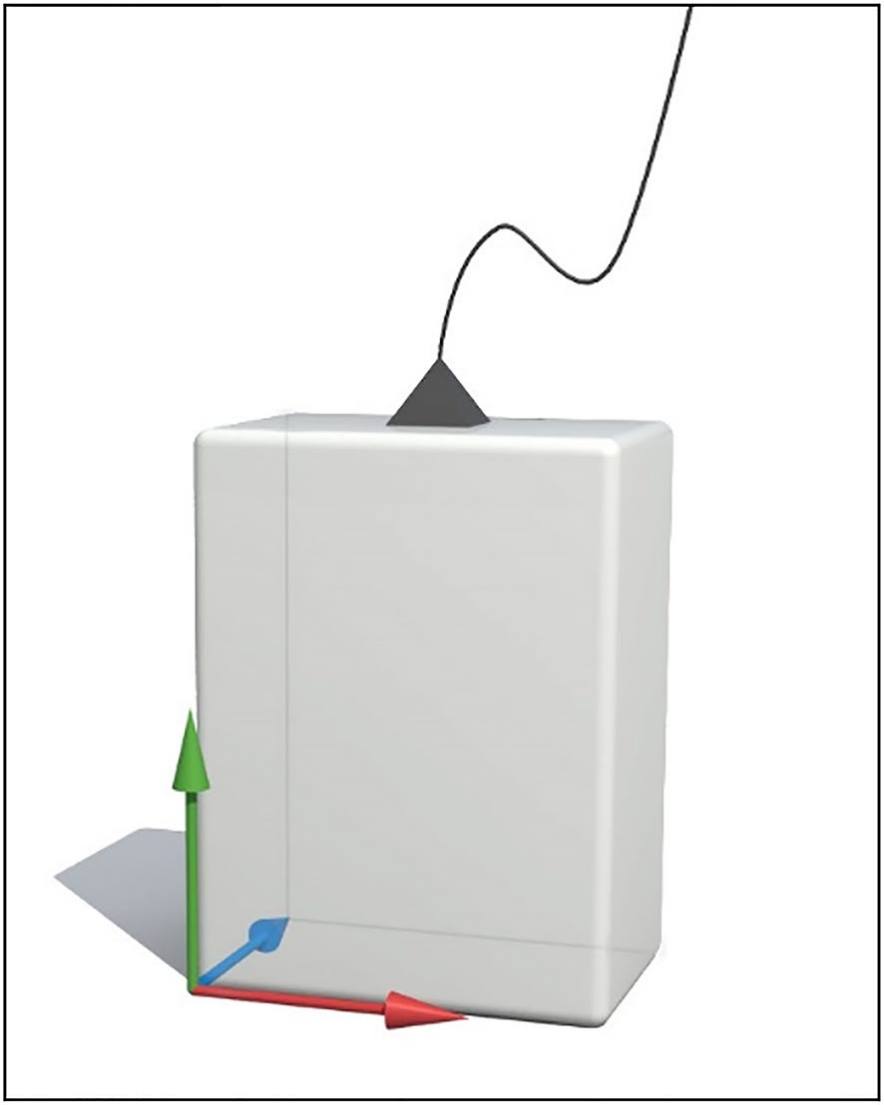
Scheme of directions of the three axis, X (*red*), Y (*green*) and Z (*blue*).

During the execution of the FRT, participants carried two inertial sensors, one placed at the level of L5–S1 (lumbar region) and the other at T7 (trunk). These were placed with the cable directed toward shoulder, so that the origin of the coordinates (X, Y, Z) (0, 0, 0) was placed at the left posterior-inferior vertex (Figure 2).

### Inertial sensors

The model InertiaCube3TM InterSense Inc. (Bedford, MA, USA) was the model of the two inertial sensors used in this study, working with a sampling frequency of 180 Hz.

The InterCube 3 is the world’s guidance smallest system (31.2 mm × 43.2 mm × 14.8 mm). It has nine sensors to ensure maximum accuracy, sensitivity and stability, covering a 360º tracking movement along three axes (Yaw, Pitch and Roll). Previous studies have demonstrated its validity and reliability in the parameterization of balance tests [27, 28].

### Procedure

Before beginning the study, participants were asked to sign the informed consent. Sociodemographic data were collected through a questionnaire and for the sample of participants who suffer stroke, who were more homogeneous, the Barthel Index (BI) (κ = 0.93 [29, 30]), the scale of impact of stroke-16 (SIS-16) (κ = 0.76 [31]) and the Canadian Neurological Scale (CNS) (ICC = 0.70–0.92 [32]) was used.

Subsequently, the FRT was explained to them and they could testing it to ensure understanding the implementation [26, 33]. Then both sensors [L5–S1 (lumbar region) and T7 (trunk)] were put in place and the functional test was carried out. Two researchers monitored the test, which was run in triplicate, and they then performed a posteriori analysis of the results independently.

The total time of data collection was the total duration of the test run for each participant and 3 s before and after the start and end of the test. It allowed the researcher to make a reference for the data analysis. Each participant performed the FRT three times. The FRT with the highest measure were used to analyze the kinematic data. In addition, all the measures (kinematic data and FRT measures) were used to calculate the reliability of the measures.

Upon execution, the kinematic data recorded by the inertial sensors were collected and were analyzed to obtain the direct variables, the time and displacement between each of the intervals, and the indirect variables, the speed and acceleration, which were subsequently calculated.

### Outcome measures

#### Direct variables

**Figure 3:**
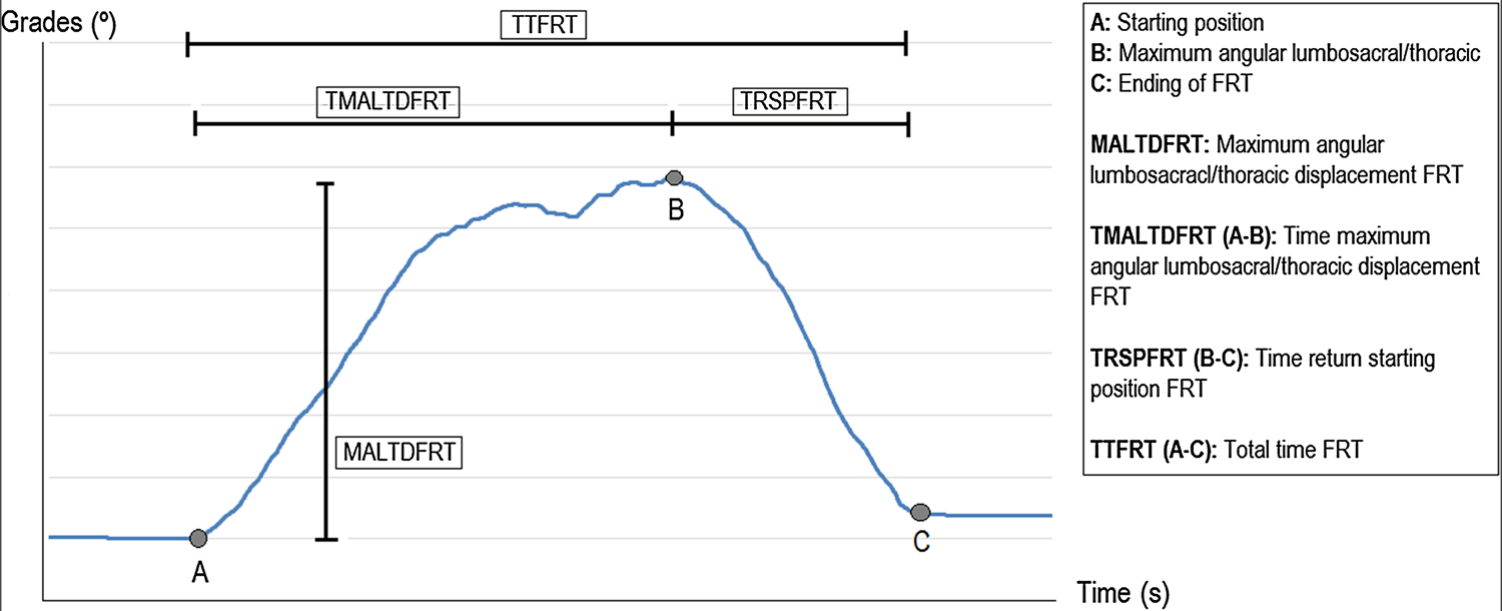
Variables extracted directly from FRT through the inertial sensor.

The variable *FRT distance* was extracted by Duncan test or FRT, which is the distance in centimeters that the subject is able to reach during the performance of the FRT. All variables mentioned below were taken from the record of the inertial sensor in the pitch axis. *Maximum angular lumbosacral/thoracic displacement FRT*: the angular variation in the pitch axis that the subject causes during the performance of the FRT. The amplitude is considered from the time the test begins until peaking imbalance before starting the return to the starting position; *time maximum angular lumbosacral/thoracic displacement FRT*: the time the subject takes to reach the peak during the execution of the FRT; *time return starting position*: the time that the subject takes to return to the starting position from reaching the peak; *total time FRT*: the time the subject takes from the start to perform the FRT until the participant comes back to the starting position (Figure 3).

#### Indirect variables

Subsequently, using the data extracted directly from the register of inertial sensors, the following variables were calculated. *Average speed FRT*: average speed at which the subject performs all the FRT; *maximum angular lumbosacral/thoracic displacement speed FRT*: the average speed at which the subject reaches the peak—from the beginning to the completion of the FRT; *starting to return position speed*: the average speed at which the subject performs the return to the starting position from the maximum peak; *average acceleration FRT*: the mean acceleration at which the subject executes all the FRT; *maximum angular lumbosacral/thoracic displacement average acceleration FRT*: the average acceleration that the subject reaches from the beginning of the test until he/her reaches the peak; *acceleration average return starting position FRT*: the average acceleration that the subject reaches from the beginning of the peak until he returns to the starting position.

To calculate the indirect variables, the following formulas were used: *speed*: displacement/time. *Acceleration*: displacement/time^2^. Both, speed and acceleration were calculated using the corresponding direct variable data: e.g., FRT average speed was calculated using the displacement and the total time used during the FRT.

In addition, the *mean* and the *standard deviation* were calculated in the maximum, the minimum and the average of the speed and the acceleration in the axis X, Y and Z in both sensors. The resultant displacement vector in the three axes (X, Y, Z) (used to analyze the displacement in the three dimensions of space simultaneously) was calculated using the formula: the square root of sum of squares ($dv = \sqrt {{X^2}\,+\,{Y^2}\,+\,{Z^2}}$).

### Data analysis

A descriptive analysis of the average range achieved in the FRT as well as all kinematic variables recorded by the two inertial sensors (trunk and lumbar region) was developed.

The normality of the variables was performed using the Kolmogórov–Smirnov (KS) test, then the lumbar region and trunk records of directly measured variables (time and displacement) and the variables obtained indirectly (speed, acceleration and resultant) were compared. The Student’s t test was used for parametric variables and Wilcoxon’s test was used for non-parametric variables. The index of significance was set at or below a value of p = 0.005.

The intrasubject and intersubject reliability of the measured variables (FRT, time and displacement) was calculated directly. For the speed and acceleration variables reliability was not calculated due to the internal consistency of their values depending on the reliability of direct variables. To calculate the reliability of the outcome variables, an analysis of the internal consistency of the measurement was conducted. Reliability was considered as a test–retest standard deviation of differences with the 95% limits of agreement [34]. To analyze the reliability the standard error measurement and intraclass correlation ratio for intrasubject and intersubject reliability were calculated. Levels of reliability were poor (ICC < 0.40), moderate (0.40 ≤ ICC < 0.60), good (0.60 ≤ ICC < 0.80), or excellent (ICC ≥ 0.80) [19].

To conduct the statistical analysis, the Statistical Package for the Social Sciences (SPSS) (version 17.0 for Windows, IL, USA) was used.

## Results

Table 1 shows the anthropometric and demographic data of the participants. Furthermore, the values of the various specific tests that each participant completes are shown. These were intended to identify the degree of involvement of the patient as a result of stroke.

**Table 1:** Descriptive and anthropometric data of the two groups analyzed

	Stroke survivors (SD)	Healthy older adults (SD)
Age (years)	72.33 (±3.97)	73.04 (±3.58)
Weight (kg)	71.26 (±14.19)	72.38 (±11.94)
Height (cm)	162.65 (±7.83)	163.11 (±7.02)
BMI (kg/m^2^)	26.69 (±3.11)	27.07 (±3.87)
Canadian Neurological Scale (0–10)	9.175 (±0.485)	–
Barthel Index (0–100)	90.25 (±4.575)	–
Stroke Index Scale_16 (0–80)	71.00 (±6.934)	–
N (woman–men)	5 (3–2)	5 (3–2)

Variables on the degree of disability caused by stroke are included in SS group.

Table 2 shows a description and comparison between groups (SS and HOA) of the kinematic variables of the FRT when the inertial sensor was placed in the trunk and the distance of the FRT. Three ranges of motion were considered based on the following points: beginning of the test, maximum angular displacement and end of the test. The variables calculated in each of these intervals were time, displacement, velocity and acceleration. Through the results shown in Table 2, the maximum, minimum, mean and standard deviation of each of these variables can be checked. Significant differences can be seen between both study groups in all the analyzed variables. Although the exercise duration was greater in HOA, the increase in both linear distance (FRT) and angle (measured in the three segments described) determines significant differences in other parameters measured indirectly.

**Table 2:** Description and differences between groups of the kinematic variables of FRT measured with the inertial sensor located at the trunk

	Strokes survivors	Healthy older adults	Mean differences (SD)
	Mean (SD)	Mean (SD)	
FRT distance (cm)	13.17 (±2.18)	36.30 (±6.04)	23.13*** (±7.92)
Trunk			
Time AB (s)	8.59 (±1.64)	9.65 (±6.23)	1.06* (±0.84)
Displacement AB (º)	12.88 (±6.90)	44.82 (±10.23)	31.94*** (±9.35)
Speed AB (º/s)	1.52 (±4.10)	6.10 (±3.05)	4.58*** (±3.28)
Acceleration AB (º/s^2^)	0.19 (±3.07)	1.04 (±0.85)	0.85*** (±0.33)
Time BC (s)	6.77 (±5.93)	4.84 (±2.12)	−1.93** (±1.04)
Displacement BC (º)	9.64 (±4.21)	48.17 (±4.23)	38.53*** (±7.30)
Speed BC (º/s)	1.48 (±0.65)	11.40 (±4.36)	9.92*** (±5.90)
Acceleration BC (º/s^2^)	0.22 (±0.19)	3.12 (±2.36)	2.9*** (±1.73)
Time AC (s)	15.61 (±4.17)	14.49 (±6.47)	−1.12* (±0.78)
Displacement AC (º)	13.58 (±7.31)	48.94 (±5.69)	35.36*** (±9.36)
Speed AC (º/s)	0.83 (±1.68)	3.87 (±1.42)	3.04*** (±2.77)
Acceleration AC (º/s^2^)	0.06 (±0.39)	0.35 (±0.22)	0.29*** (±0.08)

*A* beginning of the FRT, *B* maximum angular displacement, *C* end of the FRT.

Significance * ≤0.05, ** ≤ 0.005, *** ≤0.001.

Table 3 shows the differences between the kinematic variables collected by the inertial sensor when it was placed in the lower back. A similar behavior to that observed in the measurement of the inertial sensor in the trunk is observed. Again significant differences are observed in all kinematic variables recorded. And again, as seen in HOA, angular displacement is much higher than in SS by determining the rest of indirect variables measures (speed and acceleration).

**Table 3:** Description and differences between groups of the kinematic variables of FRT measured with the inertial sensor located at the lumbar region

	Strokes survivors	Healthy older adults	Mean differences (SD)
	Mean (SD)	Mean (SD)	
Lumbar region			
Time AB (s)	8.39 (±2.66)	9.59 (±5.12)	1.21* (±0.18)
Displacement AB (º)	7.69 (±3.81)	51.07 (±7.19)	43.38*** (±5.77)
Speed AB (º/s)	0.86 (±0.79)	42.48 (±11.27)	41.62*** (±9.35)
Acceleration AB (º/s^2^)	0.09 (±0.34)	7.31 (±5.77)	7.22*** (±4.51)
Time BC (s)	7.89 (±5.91)	4.85 (±0.86)	−3.04*** (±0.79)
Displacement BC (º)	9.48 (±3.59)	48.28 (±6.42)	38.8*** (±5.88)
Speed BC (º/s)	1.16 (±0.01)	10.24 (±2.37)	9.08*** (±2.07)
Acceleration BC (º/s^2^)	0.17 (±0.01)	2.25 (±1.00)	2.08** (±0.73)
Time AC (s)	16.4 (±3.3)	13.44 (±4.87)	−2.96** (±4.12)
Displacement AC (º)	14.81 (±6.38)	49.77 (±9.51)	34.96*** (±8.61)
Speed AC (º/s)	0.83 (±1.68)	4.11 (±1.78)	3.28*** (±1.77)
Acceleration AC (º/s^2^)	0.04 (±0.60)	0.37 (±0.25)	0.33*** (±0.19)

*A* beginning of the FRT, *B* maximum angular displacement, *C* end of the FRT.

Significance * ≤0.05, ** ≤0.005, *** ≤0.001.

**Figure 4:**
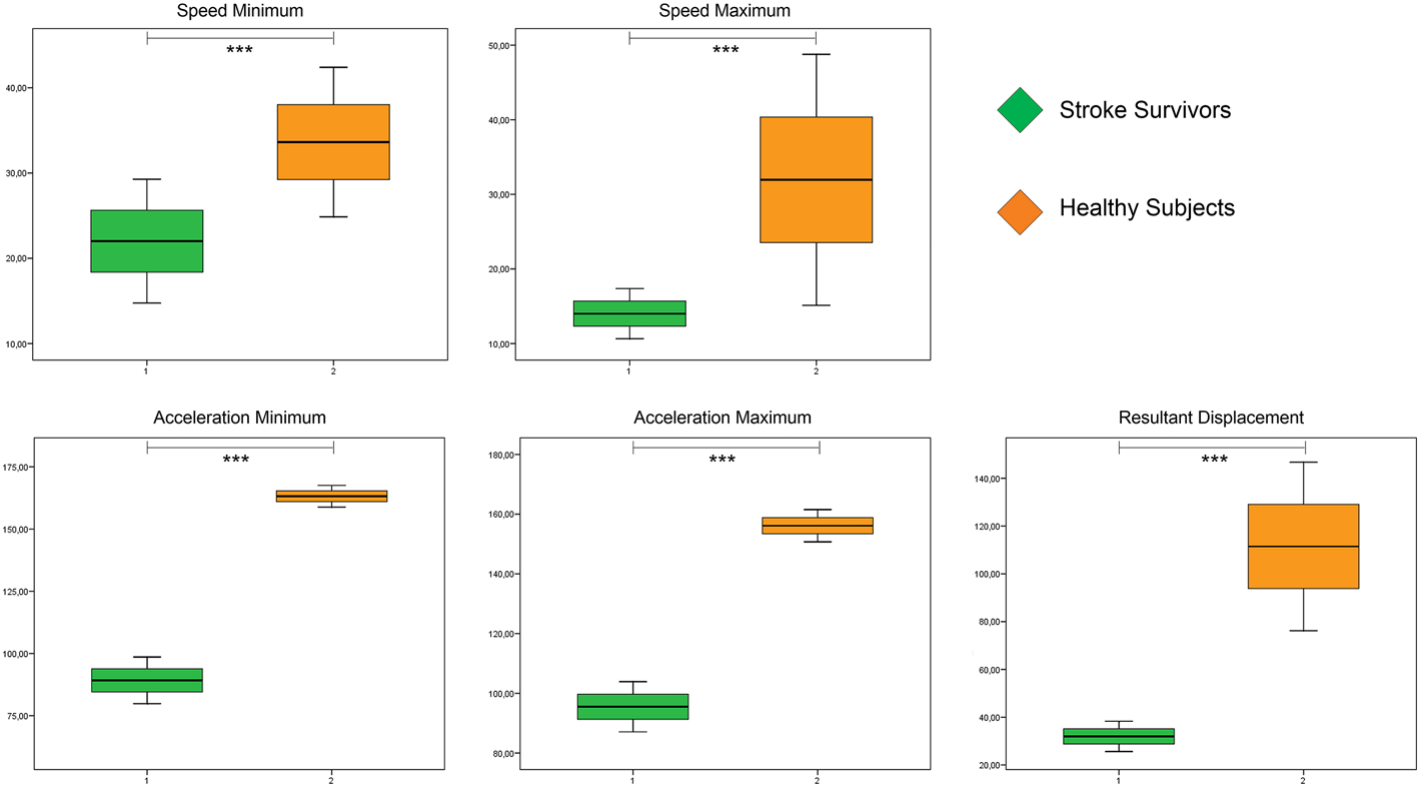
Comparison of the resulting kinematic variables b etween SS and HOA measured by trunk inertial sensor. Units of measurement: speed, º/s; acceleration, º/s^2^; displacement, º (grades). Significance * ≤0.05, ** ≤0.005, *** ≤0.001.

**Figure 5:**
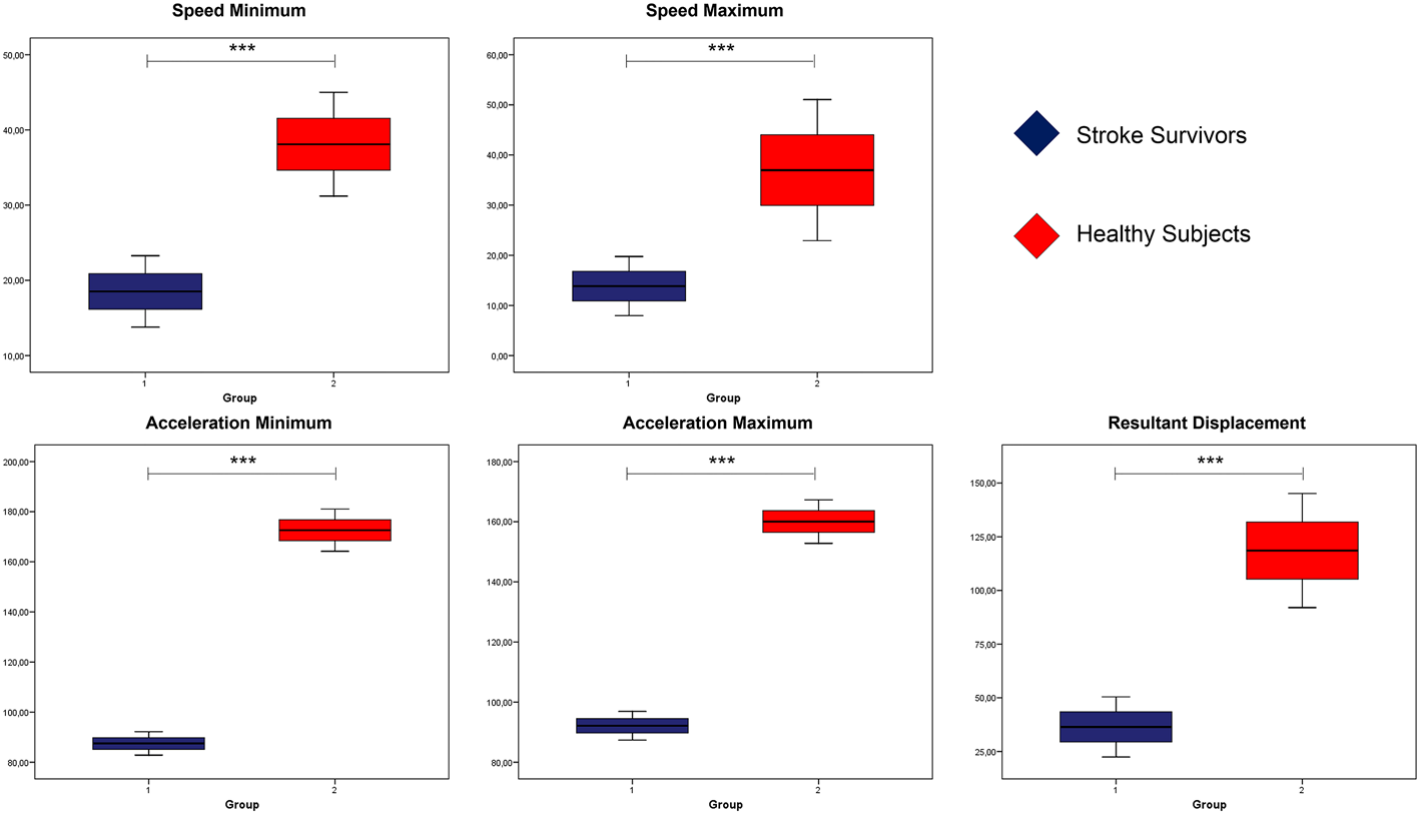
Comparison of the resulting kinematic variables between SS and HOA measured by lumbar inertial sensor. Units of measurement: speed, º/s; acceleration, º/s^2^; displacement, º (grades). Significance * ≤0.05, ** ≤0.005, *** ≤0.001.

Figures 4 and 5 show comparisons of the minimum and maximum values of the resultant of the kinematic variables. It could be observed in all compared variables (speed and minimum and maximum acceleration and the resulting displacement) there are significant differences between SS and HOA, regardless of the place where the sensor inertial was placed (trunk or lumbar region).

Table 4 shows the differences between the variables obtained indirectly (speed and acceleration) of the two groups studied (SS and HOA) after the measurements taken on both the trunk and the lumbar region differentiated axes (X, Y, Z). There are significant differences in all indirect variables analyzed. However, the differences behave differently depending on each type. In all variables that correspond to the mean, it is possible to observe, in both trunk and lumbar region, a more average velocity and acceleration on HOA group than the SS group. However, those variables that represent velocity and acceleration peaks show how the SS group has higher values than those obtained from HOA (Table 4).

**Table 4:** Trunk and lumbar region kinematic differences between groups

	Axis X			Axis Y			Axis Z		
	Mean (SD)			Mean (SD)			Mean (SD)		
	SS	HOA	Dif	SS	HOA	Dif	SS	HOA	Dif
Trunk									
Speed mean	1.20 (±1.49)	1.94 (±0.23)	0.74*** (±0.50)	15.93 (±3.27)	21.65 (±12.74)	5.72*** (±4.01)	13.48 (±7.77)	24.60 (±19.59)	11.12** (±9.01)
Speed maximum	2.19 (±0.83)	1.77 (±0.86)	0.42*** (±0.09)	43.73 (±22.79)	27.03 (±7.05)	−16.7** (±13.79)	51.69 (±30.40)	23.21 (±9.67)	−28.48*** (±12.89)
Speed minimum	0.95 (±0.67)	0.58 (±0.66)	1.53*** (±0.88)	16.51 (±8.78)	9.88 (±4.31)	−6.63*** (±3.56)	21.01 (±13.66)	9.51 (±1.45)	−11.50** (±6.11)
Acceleration mean	2.28 (±1.33)	38.67 (±70.68)	36.39*** (±26.73)	3.13 (±1.56)	7.93 (±0.72)	4.8*** (±3.05)	6.47 (±1.27)	6.71 (±2.99)	0.24*** (±0.06)
Acceleration maximum	43.84 (±69.66)	2.98 (±2.34)	−40.86** (±34.21)	8.86 (±0.76)	6.17 (±3.47)	−2.69* (±2.02)	122.89 (±68.93)	95.40 (±8.54)	−27.49*** (±17.44)
Acceleration minimum	5.09 (±1.43)	0.81 (±1.44)	−2.81** (±1.18)	2.90 (±3.07)	0.93 (±0.65)	−1.97*** (±1.71)	129.60 (±70.40)	88.88 (±9.58)	−40.72*** (±30.63)
Lumbar region									
Speed mean	1.35 (±1.30)	1.57 (±1.18)	0.22*** (±0.19)	13.72 (±4.99)	24.46 (±15.76)	10.74*** (±9.55)	11.72 (±4.39)	27.63 (±20.82)	15.91** (±6.64)
Speed maximum	1.77 (±0.86)	1.74 (±0.75)	−0.03** (±0.01)	43.73 (±22.79)	24.25 (±7.98)	−19.48** (±11.65)	51.69 (±30.39)	20.10 (±5.59)	−31.59** (±17.43)
Speed minimum	0.42 (±0.09)	0.03 (±1.56)	−0.39*** (±0.19)	19.27 (±7.18)	10.53 (±6.50)	−8.74*** (±7.37)	24.06 (±22.85)	8.38 (±1.27)	−15.68*** (±13.05)
Acceleration mean	1.27 (±2.51)	40.68 (±1.89)	39.41*** (±3.11)	1.54 (±4.50)	8.15 (±0.32)	6.61*** (±6.02)	87.36 (±4.66)	127.99 (±71.96)	40.63*** (±4.91)
Acceleration maximum	42.99 (±69.95)	2.41 (±0.90)	−40.58*** (±26.33)	8.90 (±0.91)	2.16 (±1.43)	−6.74** (±0.87)	139.57 (±75.25)	92.11 (±4.75)	−47.46*** (±5.02)
Acceleration minimum	37.32 (±68.37)	0.00 (±0.86)	−37.32*** (±19.87)	1.62 (±4.54)	0.74 (±0.93)	−0.88*** (±0.29)	11.59 (±8.93)	4.75 (±1.01)	−6.84** (±4.52)

*SS* stroke survivors, *HOA* healthy older adults, *Dif* differences between groups.

Significance * ≤0.05, ** ≤0.005, *** ≤0.001.

The intra-observer reliability of the variables measured directly shows values ranging between 0.876 (trunk time AC) and 0.916 (lumbar region displacement BC). In addition, the inter-observer reliability varies between 0.860 (trunk time AC) and 0.906 (trunk displacement BC). In turn, the reliability values of the FRT are 0.990 and 0.987 for intra-observer and inter-observer measurements respectively. The remaining reliability values analyzed in this study can be seen in Table 5.

**Table 5:** Intra-observer and inter-observer reliability of variables measured directly during Functional Reach Test

Variable	SEM (stand. error. measu.)		Intra-observer			Inter-observer		
	Strokes survivors	Healthy older adults	ICC	IC (95%)		ICC	IC (95%)	
				Min.	Max.		Min.	Max.
Trunk								
Time								
AB	0.867	2.787	0.892	0.879	0.902	0.886	0.878	0.897
BC	3.194	0.949	0.903	0.888	0.912	0.891	0.882	0.901
AC	2.329	2.893	0.876	0.869	0.890	0.860	0.852	0.871
Displacement								
AB	4.582	4.573	0.910	0.893	0.921	0.899	0.887	0.911
BC	2.364	1.893	0.913	0.902	0.921	0.906	0.893	0.914
AC	4.153	2.545	0.893	0.877	0.904	0.871	0.862	0.883
Lumbar region								
Time								
AB	1.463	2.289	0.898	0.880	0.911	0.887	0.878	0.898
BC	3.011	0.386	0.900	0.886	0.911	0.891	0.879	0.902
AC	1.851	2.178	0.881	0.870	0.898	0.869	0.858	0.877
Displacement								
AB	1.624	3.217	0.907	0.893	0.919	0.892	0.880	0.903
BC	1.840	2.870	0.916	0.905	0.922	0.902	0.890	0.911
AC	1.738	4.251	0.894	0.879	0.907	0.883	0.871	0.895
Functional Reach Test			0.990	0.983	0.997	0.987	0.979	0.994

## Discussion

After obtaining and analyzing the kinematic registration of the FRT in SS and HOA it can be stated that there are significant differences between the two study groups in all kinematic variables. The linear and angular displacement in HOA is much higher, which determines the rest of the indirect measure variables (acceleration and velocity). However, stroke survivors show higher peaks in the maximum and minimum velocity and acceleration. Moreover, the reliability of the inertial sensors as a tool for measuring kinematic variables collected during the execution of the FRT has been confirmed. All this confirms the hypothesis that was raised at the beginning of this study.

### Kinematic variable differences

After analyzing the kinematic variables obtained from the two sensors in each of the intervals into which the FRT is divided (Tables 2, 3), it can be seen that in these intervals the angular displacement was greater in the HOA group [displacement AB-lumbar region 7.69º (SS)/51.07° (HOA)]; however, they were completed at similar times for both groups [AB-lumbar region time 8.39 s (SS)/9.59 s (HOA)], which indicates that HOA conducted the test at a higher speed and greater acceleration in each of the intervals [speed AB-lumbar region 0.86º/s (SS)/42.48°/s (HOA) and acceleration AB-lumbar region 0.09º/s^2^ (SS)/7.31º/s^2^ (HOA)]. This is confirmed when it is seen that the difference is always positive for HOA with average speeds in the acceleration of each of the axes (Table 4), which shows they perform a wider, faster and accelerated movement, resulting in greater control of the movement.

However, when the maximum and minimum velocity and acceleration between the two groups were analyzed, it was found that SS values are higher than in HOA (Table 4), showing a difference of −40.86º/s^2^ and −2.81º/s^2^ in the maximum and minimum acceleration in the trunk, and −40.58º/s^2^ and −37.32º/s^2^ in the maximum and minimum acceleration in the lumbar region. The same trend is observed in the minimum and maximum acceleration and speed on both sensors and each of the axes (Table 4). All this supports the notion that SS have less motor control than HOA, which in turn denotes a lack of balance in this population.

This statement is in line with findings in other studies [1, 35] in which a kinematic registration was performed in balance tests with stroke survivors using a force platform. In these, the unbalanced area (the area of the surface describing the participant during balance control during the execution of FRT) (mm^2^/s) of stroke survivors is over twice that of HOA (43.6/15.4 mm^2^/s) [1]. In turn, it can be seen that the speed difference in the anterior–posterior and medial–lateral plane is twice as high among stroke survivors and HOA, at 12.1/6.5 and 10.1/4.7 mm/s, respectively [1]. These data reaffirm the lack of balance and postural control in stroke survivors. The movement in the Z-axis by the inertial sensors in this study cannot be compared because the force platform collected only two dimensions.

However, in a previous study in which a kinematic search was conducted during the implementation of the FRT by SS with inertial sensors located in the lumbar region and trunk [28], we note the difference between the maximum and minimum values of acceleration and velocity to the means in the register made by both sensors in each of the axes [AccMax −0.81º/s^2^/AccMin −2.98º/s^2^ and AccMed 2.17º/s^2^ (Y axis/trunk)] [28]. This proves that similar populations show high peaks of acceleration and speed in implementing the FRT, pointing again the lack of movement and postural control by SS.

### Kinematic variables

After reviewing the record made of the kinematic variables in the study of Merchan et al. [28]. In stroke survivors during the implementation of the FRT, we note that the time taken for the whole test (AC interval), the displacement achieved, and the average speed and acceleration are similar to those values of these same variables reached in SS in the present study. This justifies the reliability of inertial sensors as tools to measure movement in stroke survivors during the execution of the FRT. Time, displacement, velocity and acceleration in the AC range recorded by the sensor trunk were 15.68 s, 13.5°, 0.86°/s and 0.05°/s^2^ [28], respectively, showing consistency with the values of this study in the same interval and sensor in the lumbar region, 15.61 s, 13.58°, 0.83°/s and 0.06°/s^2^. Time, displacement, velocity and acceleration in the AC range recorded by the lumbar region sensor were 16.7 s, 14.98°, 0.89º/s and 0.05°/s^2^ [28], respectively, and are consistent with the values of the present study in the same interval and the same sensor placement, 16.4 s, 14.81°, 0.83°/s and 0.04°/s^2^.

This same trend is observed in the values of time, displacement, velocity and acceleration in the other two intervals into which the FRT is divided, from the beginning of the test to the maximum point (AB) and from the peak to the end of the test (BC), as much in the sensor located in the trunk (L_5_–S_1_) as in the sensor located in the lumbar region (T_7_).

### FRT in SS and HOA

Analyzing other studies conducting the FRT in older people with chronic stroke [2, 36, 37], we note that the values of the functional scope achieved by these SS (13.17 cm) are comparable to the average values in the FRT published in the aforementioned studies, with averages of 18.7 cm [2], 13.76 cm [36] and 18.8 cm [37], despite the difference between the average age of SS in these studies, 53.5 years/54.4 years/58.9, and the present study, 72.3 years. It could say that the disease is more prevalent on limiting balance than the age in older adults.

However, in studies such as Vernon et al. [38], in which stroke survivors suffered the stroke 1 year before the study, approximately, and had received physiotherapy treatment equilibrium, it is found that there is a difference in the range achieved in the FRT (28.50 cm) in relation to the present study (13.17 cm), despite it being a sample of similar mean age (68 and 72.33 years). The results presented in the study of Vernon et al. [37] are much closer to the results obtained by the HOA group in this study (28.50 cm [38] to 36.30 cm [present study]). So one could argue that the negative impact that have stroke victims is reversible if early intervention, in the form of an assessment, monitoring and treatment of suitable postural balance and control are carried out.

Moreover, the 36.30 cm achieved by the HOA (73.04 years) of the present study is consistent with that obtained by the subjects of previous studies: 32.2 cm [39], 30.2 cm [40], 32 cm [41] and 36.79 cm [42]. The average age of these groups of subjects was practically the same as those in this study: 70.3, 77, 74.14 and 65.3 years.

### Reliability of measures

The reliability results obtained in this study show an intra-observer reliability of 0.876–0.913 (trunk) and 0.881–0.916 (lumbar region) and inter-observer reliability of 0.860–0.906 (trunk) and 0.869–0.902 (lumbar region) (Table 5), so it can be confirmed that the levels of reliability are excellent (ICC > 0.80) [18, 19]. Furthermore, they are consistent with previous studies consulted: intra: 0.80–0.94 and inter: 0.79–0.90 (trunk) [20], intra: 0.835–0.891 and inter: 0.831–0.883 (trunk) and intra: 0.829–0.878 and inter: 0.821–0.875 (lumbar region) [28], intra: 0.68–0.95 [43] and intra: 0.78–0.94 [44].

In analyzing the reliability of the measures of the functional range it can be seen as in the FRT that stroke survivors have higher levels of reliability to 0.98 [ICC: 0.990 (0.983–0.997) and 0.987 (0.979–0.989) for intra-and inter-observer reliability]. These levels are consistent with those observed in the previous study that performed the kinematic record with two inertial sensors in the FRT and stroke survivors [ICC: 0.987 (intra) and 0.983 (inter-observer)] [28].

### Strengths and weaknesses

As this is a pilot study the sample consisted of 10 participants, 5 HOA and 5 stroke survivors. Therefore, it would be necessary to extend the sample of participant up to 40 participant, approximately, as performed previous studies with similar characteristics [36, 37]. Registration of the kinematic variables has wide applicability in both basic research and clinical practice. Furthermore, comparison of the data obtained allows the characteristics of movement and postural control in people who have suffered stroke to be met reliably.

## Conclusions

The SS show less functional reach, a narrower, slower and less accelerated movement, but with higher peaks of acceleration and speed when they are compared with HOA. This shows some imprecision in movement and lack of postural control, which can lead to a greater imbalance and thus an increased risk of falls in stroke survivors.

The reliability and validity shown by the inertial sensors, combined with their low cost and portability, make them ideal tools for identifying the differences in kinematic variables among SS and HOA, both lumbar region and trunk. This fact, and the results obtained in this study, will enable the characteristics of movement and gestures of SS to be assessed more precisely in clinical practice, allowing interventions to be performed and tracking to be more accurate in terms of postural control and balance, and, therefore, a greater risk prevention of falls.

## Abbreviations

FRT: Functional Reach Test
SS: stroke survivors
HOA: healthy older adults
BI: Barthel Index
KS: Kolmogórov–Smirnov
